# Cross-Cyclotrimerization with Two Nitriles as a Synthetic Pathway to Unsymmetrically 3,3’-Disubstituted bis(Tetrahydroisoquinolines)

**DOI:** 10.3390/molecules14082918

**Published:** 2009-08-10

**Authors:** Aneta Kadlčíková, Martin Kotora

**Affiliations:** 1 Department of Organic and Nuclear Chemistry, and Center for Structural and Synthetic Application of Transition Metal Complexes, Faculty of Science, Charles University in Prague, Hlavova 8, 128 43 Praha 2, Czech Republic; 2 Institute of Organic Chemistry and Biochemistry, Academy of Sciences of the Czech Republic, Flemingovo nám. 2, 166 10 Praha 6, Czech Republic

**Keywords:** cyclotrimerization, cobalt, microwave heating, pyridines

## Abstract

Microwave assisted CpCo(CO)_2_ catalyzed cross-cyclotrimerizations of 1,7,9,15-hexadecatetrayne with two different nitriles to give unsymmetrically substituted bis(tetrahydroisoquinolines) was studied. The reaction proceeded with a range of alkyl and aryl nitriles with reasonable isolated yields.

## 1. Introduction

Lewis base catalyzed processes constitute a large group of reactions that has been extensively used in organic synthesis and especially in asymmetric catalysis [1]. Among Lewis bases that have recently attracted considerable attention are pyridine *N*-oxides and their congeners. For example, they are able to strongly activate halosilanes via Lewis base-acid interaction to such an extent that halosilanes react with various functional groups. In this respect, enantioselective catalytic allylation of aldehydes have been intensively studied [2,3,4,5,6,7,8]. A number of various *N*-oxides [9,10,11,12,13,14,15,16,17,18,19,20], *N,N*-dioxides [21,22,23,24,26,27] and even *N,N,N*-trioxides [28] based on heterocyclic compounds with pyridine ring have been synthesized for this purpose. 

We have recently demonstrated that microwave irradiation-enhanced Co-complex catalyzed [2+2+2]-cross-cyclotrimerization of diynes with nitriles can be conveniently used for simple and expedient synthesis of various bipyridines. Their oxidation, usually by MPCBA, and resolution into enantiomers yielded the corresponding chiral bipyridine *N,N-*dioxides [29,30,31,32,33]. The compounds catalyzed asymmetric allylation of aldehydes in up to 87% ee. In our last report we showed that the cyclotrimerization of 1,7,9,15-hexadecatetrayne with two different nitriles, benzonitrile and (*R*)-tetrahydrofurancarbonitrile, furnished unsymmetrically substituted bis(tetrahydroisoquinoline) in 28% isolated yield, along with the symmetric derivative (20% isolated yield). Its further oxidation afforded two diastereoisomeric bis(tetrahydroisoquinoline) *N,N*’-dioxides that catalyzed enantioselective allylation of aldehydes [34]. This result provided us with impetus to study potential application of cobalt-complex catalyzed cross-cyclotrimerization of 1,7,9,15-hexadecatetrayne (**1**) with various nitriles as a pathway to unsymmetrically substituted bis(tetrahydroisoquinolines).

## 2. Results and Discussion

The microwave assisted cyclotrimerization of alkynes with nitriles offers several advantages in comparison with standard thermal conditions. Among these are, for example, reduced reaction times from hours to minutes, reactants are thus exposed to high reaction temperatures for shorter periods of time reducing possibility of thermal decomposition, and also the use of lower boiling point solvents such as THF, which allows easier separation of products [35,36,37,38,39,40].

**Scheme 1:**
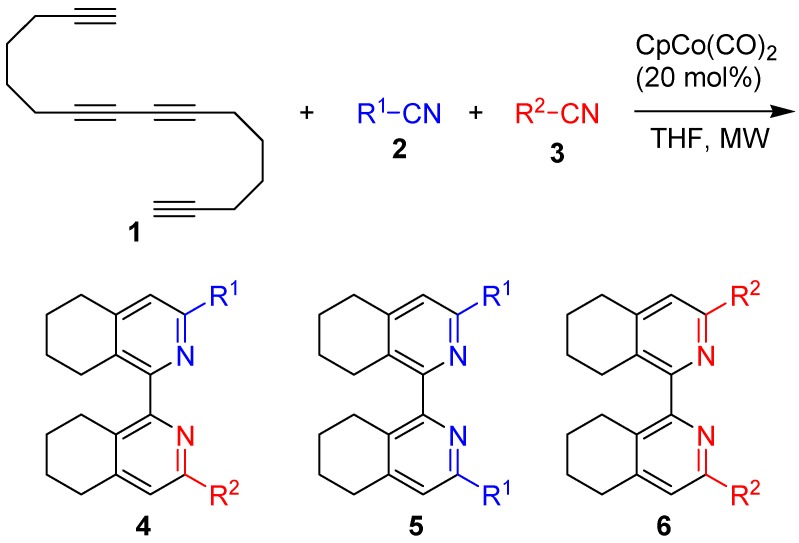
Co-cyclotrimerization of tetrayne **1** with nitriles **2** and **3**.

For the study of cross-cyclotrimerization of 1,7,9,15-hexadecatetrayne (**1**) with various nitriles the previously used conditions were applied: CpCo(CO)_2_ (20 mol%), nitrile R^1^-CN (1 equiv.), nitrile R^2^-CN (1-20 equiv.), THF as solvent. The reactions were carried out in microwave reactor (microwave irradiation 300 W, reaction time 25 min). A series of cross-cyclotrimerization reactions between two different nitriles with 1,7,9,15-hexadecatetrayne (**1**) in various molar ratios was carried out with varied success (Scheme 1). 

In all cases the desired products of cross-cyclotrimerization of **1** with two different nitriles **2** and **3** – unsymmetrically substituted bis(tetrahydroisoquinolines) **4** – were formed. The reaction was always accompanied by the formation of varying amounts of symmetrically substituted bis(tetrahydr-oisoquinolines) **5** and/or **6**, the products of cyclotrimerization of **1** with **2** or **3**. Some of the most representative examples are given in Table 1. 

**Table 1:** Cross-cyclotrimerization of tetrayne **1** with two nitriles **2** and **3**.

Entry	R^1^-CN	R^2^-CN		R^1^/R^2 a^	Yield (%)^b^ 4	Yield (%)^b^ 5	Yield (%)^b^ 6
1	C_6_H_5_	CH_3_	**3a**	1/1	**4a**, 10	**5a**, 30	0
2				1/10	25	10	0
3				1/20	25	10	0
4	C_6_H_5_	4-MeOC_6_H_4_	**3b**	1/1	**4b**, 23	**5a**, 15	0
5				1/10	28	10	0
6	C_6_H_5_	2-C_5_H_4_N^e^	**3c**	1/1	**4c**, 20	**5a**, 10	0
7				1/5	28	2	0
8	C_6_H_5_	THF*^c^	**3d**	1/1	**4d**, 28	**5a**, 10	0
9				1/10	24	10	0
10	CH_3_	3,4,5-(MeO)_3_C_6_H_4_	**3e**	1/1	**4e**, 20	traces	**6e**, 9
11				10/1	28	0	5
12	CH_3_	4-CF_3_C_6_H_4_	**3f**	1/1	**4f**, 12	0	**6f**, 30
13				10/1	20	0	15
14	CH_3_	4-ClC_6_H_4_	**3g**	1/1	**4g**, 22	0	**6g**, 10
15				10/1	27	0	5
16	CH_3_	THF*^c^	**3d**	1/1	**4h**, 16^d^	0	**6h**, 16^d^
17				10/1	16^d^	**5b**, 16^d^	3^d^

^a^ Molar ratio. ^b^ Isolated yields. ^c^ (*R*)-tetrahydrofuran-2-yl. ^d^ ^1^H-NMR yields. ^e^ 2-pyridyl.

Thus the cross-cyclotrimerization of benzonitrile (**2**) with acetonitrile (**3a**, 1/1 **2/3a** ratio) gave preferentially symmetrical product **5a** (Entry 1), the change of the nitrile ratio to 1/10 resulted in the preferential formation of **4a** (25% isolated yield) which was accompanied by a small amount of **5a** (10%) (Entry 2). The formation of **6a** was not detected. Further change of the nitrile ratio did not affect the reaction outcome (Entry 3). The analogical results were obtained also in other cases. Thus cross-cyclotrimerization of benzonitrile (**2**) and 4-methoxybenzonitrile (**3b**, 1/10 **2/3b** ratio) gave **4b** in 28% isolated yield along with 10% of **5a** (Entry 5). The formation of **4c** was achieved in 28% isolated yield (1/5 **2/3c** ratio) and **5a** was formed in just 2% yield (Entry 7). Finally benzonitrile (**2**) and (*R*)-tetrahydrofurancarbonitrile (**2d**) were cross-cyclotrimerized to yield the corresponding **4d** in 28% isolated yield. Unlike in the previous cases, the best result was obtained when both nitriles were used in 1/1 molar ratio (compare Entries 8 and 9). In none of the cases was the formation of **6a-6d** detected.

Then combination of acetonitrile (**3a**) and other nitriles was also studied. The best results for cyclotrimerization of **3a** with nitriles **3e-3g** giving the highest yields of the corresponding bis(tetrahydroisoquinolines) **4e**-**4g** (28, 20, and 27% yields, respectively) occurred when the molar ratios of **3a/3e**, **3a/3f**, and **3a/3g** were 10/1 (Entries 11, 13, and 15). On the other hand, for the reactions of **3a** with nitrile **3d** the optimal ratio of **3a/3d** was 1/1 (Entry 16). The formation **5b** was not observed under these conditions.

It should be emphasized that the cross-cyclotrimerization proceeded only under microwave irradiation. Carrying out the reaction under standard thermal condition (130 °C, 24-48 h) or attempts to facilitate the cyclotrimerization by visible light irradiation gave rise to intractable reaction mixtures. 

Attempts to carry out the cyclotrimerization sequentially, i.e. to cyclotrimerize tetrayne **1** with one nitrile, isolate the expected intermediate (1-diynyltetrahydroisoquinoline), and then to carry out the second cyclotrimerization with another nitrile, also failed. The first reaction under the above mentioned conditions usually proceeded with low selectivity for the monocyclotrimerization (only up to 30%) and low yields (~10%), regardless of the nitrile used, making the whole process uneconomical. Carrying out the monocyclotrimerization under less forcing conditions by using another catalytic system, e.g. CoCl_2_·6H_2_O, Zn, dppe [41,42], did not meet the expectations either. Although the reaction proceeded exclusively to yield 1-diynyltetrahydroisoquinolines, the yields did not exceed in 10%. The use of catalysts based on other transition metals (Rh, Ru) did not promote the cyclotrimerization.

## Experimental

### General

All solvents, unless otherwise stated, were used as obtained. THF was distilled from sodium and benzophenone under Ar. All other reagents were obtained from commercial sources. ^1^H- and ^13^C- NMR spectra were recorded on a Varian UNITY 300 (^1^H at 300 MHz, ^13^C at 75 MHz) as solutions in C_6_D_6_. Chemical shifts are given in δ-scale, coupling constants *J* are given in Hz. Mass spectra were recorded on a LTQ Orbitrap XL. Infrared spectra were recorded on a FTIR Nicolet avatar Drift KBr and are reported in wave numbers (cm^-1^). Fluka 60 silica gel was used for flash chromatography. TLC was performed on silica gel 60 F_254_-coated aluminum sheets (Merck). All reactions were carried out under an argon atmosphere using flasks or in microwave reactor Biotage Initiator. Hexadeca-1,7,9,15-tetrayne (**1**) was prepared according to the previously reported method [43].

### General procedure for cross-cyclotrimerization

R^1^CN (0.48 mmol), R^2^CN (4.8 mmol) and CpCo(CO)_2_ (18 mg, 0.1 mmol) were added under atmosphere of argon to a solution of hexadeca-1,7,9,15-tetrayne (**1**, 100 mg, 0.48 mmol) in dry and degassed THF (3 mL) contained in a vial. Then the vial was placed into the microwave oven and irradiated for 25 min (300 W) (during the process temperature and pressure reached 180 °C and 20 barr, respectively). Unreacted nitriles were then removed under reduced pressure and column chromatography of the residue on silica gel using the indicated eluent systems yielded the products.

*5,6,7,8-Tetrahydro-1-(5,6,7,8-tetrahydro-3-methylisoquinolin-1-yl)-3-phenylisoquinoline* (**4a**). Eluent: 3/1 hexane/EtOAc; yield: 40 mg (25%) of the title compound as a viscous colorless liquid and 30 mg (10%) of symmetrical benzonitrile substituted product **5a**. Compound **4a**: ^1^H-NMR: δ 1.44-1.49 (m, 8H), 2.44-2.48 (m, 4H), 2.51 (s, 3H), 2.61-2.66 (m, 4H), 6.59 (s, 1H), 7.19 (s, 1H), 7.24-7.29 (m, 3H), 8.17-8.20 (m, 2H); ^13^C-NMR: δ 23.2 (2C), 23.9, 24.0, 24.8, 26.8, 27.0, 30.2, 30.5, 120.4, 123.3, 127.9 (2C), 128.2, 128.5, 129.2, 129.4, 130.8, 141.0, 147.4, 148.0, 154.0, 154.7, 158.7, 159.3; IR (THF) ν 696, 1069, 1430, 1590, 1722, 2858, 2933, 3085 cm^-1^; ESI-MS m/z (% relative intensity) 355 (M^+^, 100), 377 (15); HR-ESI calculated for C_25_H_26_N_2_ 355.2169 found 355.2169.

*5,6,7,8-Tetrahydro-1-(5,6,7,8-tetrahydro-3-phenylisoquinolin-1-yl)-3-(4-methoxyphenyl)iso-quinoline* (**4b**). Eluent: 3/1 hexane/EtOAc; yield: 60 mg (28%) of the title compound as a viscous colorless liquid and 20 mg (10%) of symmetrical benzonitrile substituted product **5a**. Compound **4b**: ^1^H-NMR: δ 1.48-1.50 (m, 8H), 2.51-2.53 (m, 4H), 2.69-2.71 (m, 4H), 3.31 (s, 3H), 6.91-6.93 (m, 2H), 7.18-7.20 (m, 1H), 7.19-7.21 (m, 1H); 7.27-7.32 (m, 3H), 8.21-8.24 (m, 4H); ^13^C-NMR: δ 23.2, 23.3, 23.9, 24.0, 27.1 (2C), 30.5 (2C), 55.5, 115.0, 119.8, 120.5, 127.9 (2C), 129.2 (4C), 129.5 (2C), 130.3, 131.1, 133.6, 141.0, 148.1 (2C), 153.9, 154.0, 159.0, 159.2, 161.4; IR (THF) ν 694, 835, 1030, 1172, 1247, 1307, 1416, 1451, 1514, 1550, 1589, 1608, 2931 cm^-1^; ESI-MS m/z (% relative intensity) 447 (M^+^, 100), 448 (36), 469 (8), 893 (50), 915 (50), 916 (35), 917 (15); HR-ESI calculated for C_30_H_30_N_2_O 447.2431 found 447.2426.

*5,6,7,8,-Tetrahydro-1-(5,6,7,8-tetrahydro-3-phenylisoquinolin-1-yl)-3-(pyridin-2-yl)isoquino-line* (**4c**). Eluent: 1/1 hexane/EtOAc; yield: 55 mg (28%) of the title compound as a viscous colorless liquid and 4 mg (2%) of symmetrical benzonitrile substituted product **5a**. Compound **4c**: ^1^H-NMR: δ 1.44-1.46 (m, 8H), 2.43-2.64 (m, 8H), 6.84-6.85 (m, 1H), 7.18-7.21, (m, 3H), 7.26-7.32 (m, 2H), 8.19-8.29 (m, 3H), 8.57-8.58 (m, 1H), 9.52 (s, 1H); ^13^C-NMR: δ 22.0, 22.2, 22.8, 22.9, 26.1, 29.3, 29.4, 30.0, 119.7, 123.1, 126.9 (2C), 128.3 (2C), 128.5, (2C), 130.0, 130.8, 133.7, 135.0, 139.8, 147.3, 147.5, 148.4, 149.6, 150.5, 153.1, 157.7, 158.4; IR (THF) ν 689, 751, 806, 1024, 1160, 1430, 1588, 2855, 2922, 3036 cm^-1^; ESI-MS m/z (% relative intensity) 418 (M^+^, 100), 440 (61); HR-ESI calculated for C_29_H_27_N_3_ 418.2269, found 418.2278.

*3-[(R)-Tetrahydrofuran-2-yl]-3’-phenyl-(5,5’,6,6’,7,7’,8,8’-octahydro-l,l’-biisoquinoline)* (**4d**). Eluent: 5/1 hexane/EtOAc; yield: 56 mg (28%) of the title compound as a viscous colorless liquid and 20 mg (20%) of symmetrical product **5a**. Compound **4d**: ^1^H-NMR: δ 1.36-1.68 (m, 11H), 2.08-2.14 (m, 1H), 2.19-2.26 (m, 1H), 2.46-2.68 (m, 7H), 3.74-3.80 (m, 1H), 3.95-4.0 (m, 1H), 5.23 (t, *J*= 7Hz; 1H), 7.15-7.19 (m, 1H), 7.25-7.29 (m, 3H), 7.4 (s, 1H), 8.16-8.19 (m, 2H); ^13^C-NMR: δ 23.2 (2C), 24.0 (2C), 26.7, 27.0, 27.1, 30.4, 30.5, 34.0, 69.5, 82.4, 120.3, 120.5, 128.2, 128.6 (2C), 129.2 (2C), 130.4, 131.0, 140.9, 148.0, 148.1, 153.9, 158.4, 159.1, 160.1; IR (THF) ν 699, 961, 1069, 1432, 1722, 2877, 2937, 3292 cm^-1^; ESI-MS m/z (% relative intensity) 411 (M^+^, 100), 412 (31), 433 (27); HR-ESI calculated for C_28_H_30_N_2_O+H^+^ 411.2431, found 411.2421.

*5,6,7,8-Tetrahydro-1-(5,6,7,8-tetrahydro-3-(3,4,5-trimethoxyphenyl)isoquinolin-1-yl)-3-methyl-iso-quinolne* (**4e**). Eluent: 3/1 hexane/EtOAc; yield: 60 mg (28%) of the titled compound as a viscous colorless liquid and 2 mg (5%) of symmetrical 3,4,5-trimethoxybenzonitrile substituted product **6e**. Compound **4e**: ^1^H-NMR: δ 1.46-1.51 (m, 8H), 2.42-2.65 (m, 11H), 3.42 (s, 6H), 3.88 (s, 3H), 6.56 (s, 1H), 7.42 (s, 1H), 7.53 (s, 2H); ^13^C-NMR: δ 23.3, 23.9, 24.0, 24.7, 26.8, 26.9, 30.2, 30.5, 56.6 (2C), 61.2, 106.1, 120.3, 123.4, 128.6 (2C), 129.0 (2C), 130.5, 136.4, 141,4, 147.4, 148.0, 154.3, 155.0, 155.1, 158.7, 159.3; IR (THF) ν 853, 1068, 1127, 1228, 1430, 1554, 2858, 2934, 1584, 1721 cm^-1^; ESI-MS m/z (% relative intensity) 445 (M^+^, 100), 467 (33); HR-ESI calculated for C_28_H_32_N_2_O_3_ 445.2486 found 445.2476.

*1-(3-(4-(Trifluoromethyl)phenyl)-5,6,7,8-tetrahydroisoquinolin-1-yl)-5,6,7,8-tetrahydro-3-methyl-isoquinoline* (**4f**). Eluent: 3/1 hexane/EtOAc; yield: 40 mg (20%) of this product as a viscous colorless liquid and 30 mg (15%) of symmetrical 4-(trifluoromethyl)benzonitrile substituted product **6f**. Compound **4f**: ^1^H-NMR: δ 1.46-1.50 (m, 8H), 2.45-2.48 (m, 4H), 2.51 (s, 3H), 2.58-2.64 (m, 4H), 6.60 (s, 1H), 7.16 (s, 1H), 7.47 (m, 2H), 8.00 (m, 2H); ^13^C-NMR: δ 23.1, 23.2, 23.7, 23.8, 24.0, 24.7, 26.8, 27.0, 30.1, 30.4, 32.6, 120.8, 123.5, 126.3, 126.4, 127.7, 128.1, 129.2, 132.0, 144.1, 148.0, 148.3, 152.2, 154.8, 158.3, 159.6; IR (THF) ν 848, 1015, 1068, 1123, 1161, 1323, 1432, 1591, 2858, 2928 cm^-1^; ESI-MS m/z (% relative intensity) 423 (M^+^, 100), 445 (58); HR-ESI calculated for C_26_H_25_N_2_F_3_ 445.2486 found 445.2476.

*1-(3-(4-Chlorophenyl)-5,6,7,8-tetrahydroisoquinolin-1-yl)-5,6,7,8-tetrahydro-3-methylisoquinoline* (**4g**). Eluent: 3/1 hexane/EtOAc; yield: 50 mg (27%) of this product as a viscous colorless liquid and 9 mg (5%) of symmetrical 4-chlorobenzonitrile substituted product **6g**. Compound **4g**: ^1^H-NMR: δ 1.43-1.49 (m, 8H), 2.44-2.47 (m, 4H), 2.51 (s, 3H), 2.57-2.63 (m, 4H), 6.59 (s, 1H), 7.12 (s, 1H), 7.21-7.24 (m, 2H), 7.89-7.92 (m, 2H); ^13^C-NMR: δ 23.2, 23.3, 23.9, 24.0, 24.7, 26.8, 26.9, 30.1, 30.4, 120.2, 123.4, 128.8 (2C), 129.2 (2C), 129.6, 131.2, 135.3, 139.3, 147.5, 148.1, 152.6, 154.8, 158.5, 159.4; IR (THF) ν 834, 1012, 1304, 1431, 1492, 1592, 2857, 2932 cm^-1^; ESI-MS m/z (% relative intensity) 389 (M^+^, 100), 391 (36); ESI-MS m/z (% relative intensity) 389 (M^+^, 100), 391 (36); HR-ESI calculated for C_25_H_25_N_2_Cl 389.1779 found 389.1779.

*5,6,7,8-Tetrahydro-1-(5,6,7,8-tetrahydro-3-methylisoquinolin-1-yl)-3-(tetrahydrofuran-2-yl)-isoquinoline* (**4h**). Eluent: EtOAc; yield: 52 mg (32%) of the mixture of this product and the symmetrical (*R*)-tetrahydrofurancarbonitrile substituted product **6h** in the ratio of 1:1. Compound **4h**: ^1^H-NMR: δ 1.36-1.66 (m, 12H), 2.01-2.10 (m, 2H), 2.18-2.29 (m, 2H), 2.38-2.50 (m, 7H), 3.74-3.79 (m, 1H), 3.91-3.97 (m, 1H), 5.15-5.19 (m, 1H), 6.56 (s, 1H), 7.36 (s, 1H); ^13^C-NMR: δ 22.9, (2C), 23.7, 24.3, 26.3, 26.5, 26.6, 29.8, 30.1, 30.5, 33.6, 69.1, 82.0, 119.8, 199.9, 129.8, 130.0, 147.5, 147.6, 158.0, 158.3, 159.7, 159.8.

## Conclusions

In conclusion, we have demonstrated that CpCo(CO)_2_ catalyzed cross-cyclotrimerization of 1,7,9,15-hexadecatetrayne with two different nitriles under microwave irradiation can be a convenient method for synthesis of unsymmetrically substituted bis(tetrahydroisoquinolines). Although the isolated yields of the unsymmetrical products did not exceed 30%, this shortcoming is counterweighed by the simplicity and expeditiousness of the cross-cyclotrimerization method and also by the fact that sequential two step cyclotrimerization cannot be applied due to low selectivity and thermal instability of the starting tetrayne. Last but not least, it also should taken into the account that during two catalytic cycles six new bonds are formed. 
